# Prevention of mother-to-child transmission of hepatitis B virus in antenatal care and maternity services, Mozambique

**DOI:** 10.2471/BLT.20.281311

**Published:** 2021-12-02

**Authors:** Anne Loarec, Aude Nguyen, Lucas Molfino, Mafalda Chissano, Natercia Madeira, Barbara Rusch, Nelly Staderini, Aleny Couto, Iza Ciglenecki, Natalia Tamayo Antabak

**Affiliations:** a Médecins Sans Frontières – Mozambique, Av. Tomas Nduda 1489, Maputo, Mozambique.; b Operational Centre, Médecins Sans Frontières, Geneva, Switzerland.; c Ministry of Health of Mozambique, Maputo, Mozambique.

## Abstract

**Objective:**

To pilot an intervention on the prevention of mother-to-child transmission (PMTCT) of hepatitis B virus (HBV) in an antenatal care and maternity unit in Maputo, Mozambique, during 2017–2019.

**Methods:**

We included HBV in the existing screening programme (for human immunodeficiency virus (HIV) and syphilis) for pregnant women at their first consultation, and followed mother–child dyads until 9 months after delivery. We referred women who tested positive for hepatitis B surface antigen (HBsAg) for further tests, including hepatitis B e antigen (HBeAg) and HBV viral load. According to the results, we proposed tenofovir for their own health or for PMTCT. We administered birth-dose HBV vaccine and assessed infant HBV status at 9 months.

**Findings:**

Of 6775 screened women, 270 (4.0%) were HBsAg positive; in those for whom data were available, 24/265 (9.1%) were HBeAg positive and 14/267 (5.2%) had a viral load of > 200 000 IU/mL. Ninety-eight (36.3%) HBsAg-positive women were HIV coinfected, 97 of whom were receiving antiretroviral treatment with tenofovir. Among HIV-negative women, four had an indication for tenofovir treatment and four for tenofovir PMTCT. Of 217 exposed liveborn babies, 181 (83.4%) received birth-dose HBV vaccine, 160 (88.4%) of these < 24 hours after birth. At the 9-month follow-up, only one out of the 134 tested infants was HBV positive.

**Conclusion:**

Our nurse-led intervention highlights the feasibility of integrating PMTCT of HBV into existing antenatal care departments, essential for the implementation of the triple elimination initiative. Universal birth-dose vaccination is key to achieving HBV elimination.

## Résumé

**Objectif:**

Piloter une action de prévention d'une transmission de la mère à l'enfant (PTME) du virus de l'hépatite B (VHB) dans une maternité et unité de soins prénataux à Maputo, au Mozambique, entre 2017 et 2019.

**Méthodes:**

Nous avons inclus le VHB dans le programme existant visant à dépister le virus de l'immunodéficience humaine (VIH) et la syphilis chez les femmes enceintes lors de leur première consultation. Nous avons également suivi les dyades mère-enfant jusqu'à 9 mois après l'accouchement. Nous avons orienté les femmes positives à la présence de l'antigène de surface (HBsAg) de l'hépatite B vers d'autres tests, dont ceux détectant l'antigène e (HBeAg) et la charge virale du VHB. En fonction des résultats, nous avons proposé un traitement au ténofovir pour leur propre santé ou pour la PTME. Enfin, nous avons administré un vaccin contre le VHB à la naissance et évalué le statut sérologique du nourrisson à neuf mois.

**Résultats:**

Sur 6775 femmes dépistées, 270 (4,0%) étaient positives au HBsAg; parmi celles pour qui des données étaient disponibles, 24/265 (9,1%) étaient positives au HBeAg et 14/267 (5,2%) présentaient une charge virale supérieure à 200 000 UI/ml. Le VIH a en outre été détecté chez 98 (36,3%) femmes positives au HBsAg, 97 d'entre elles faisant l'objet d'un traitement antirétroviral au ténofovir. Parmi les femmes négatives au VIH, quatre avaient reçu des indications de traitement au ténofovir et quatre s'étaient vu conseiller le ténofovir dans le cadre d'une PTME. Sur les 217 bébés exposés, 181 (83,4%) ont été vaccinés contre le VHB à la naissance, 160 (88,4%) d'entre eux moins de 24 heures après leur venue au monde. Lors de la visite de suivi neuf mois après la naissance, seul un des 134 nourrissons testés était positif au VHB.

**Conclusion:**

Notre action dirigée par le personnel infirmier démontre qu'il est possible d'intégrer la PTME du VHB dans les départements de soins prénataux existants, une démarche essentielle à la mise en œuvre de l'initiative de triple élimination. La vaccination universelle à la naissance est cruciale pour éradiquer le VHB.

## Resumen

**Objetivo:**

Realizar una intervención piloto sobre la prevención de la transmisión maternofilial (PTMF) del virus de la hepatitis B (VHB) en una unidad de asistencia prenatal y maternidad en Maputo, Mozambique, entre 2017 y 2019.

**Métodos:**

Se incluyó el VHB en el programa existente de cribado del virus de la inmunodeficiencia humana (VIH) y de la sífilis para las embarazadas en su primera consulta, y se realizó un seguimiento de las parejas maternofiliales hasta nueve meses después del parto. Se derivó a las mujeres que dieron positivo en la prueba del antígeno de superficie del virus de la hepatitis B (AgHBs) para que se sometieran a otras pruebas, como el antígeno *e* del virus de la hepatitis B (AgHBe) y la concentración vírica del VHB. Según los resultados, se propuso tenofovir como medida sanitaria o para la PTMF. Por último, se administró la vacuna contra el VHB al nacer y se evaluó el estado serológico del VHB en los lactantes a los nueve meses.

**Resultados:**

De las 6775 mujeres analizadas, 270 (4,0 %) dieron positivo en la prueba del AgHBs; entre las que disponían de datos, 24/265 (9,1 %) eran positivas en la prueba del AgHBe y 14/267 (5,2 %) tenían una concentración vírica superior a 200 000 UI/ml. Noventa y ocho (36,3 %) mujeres con AgHBs positivo estaban coinfectadas por el VIH, 97 de las que recibían tratamiento antirretrovírico con tenofovir. Entre las mujeres con VIH negativo, cuatro tenían indicación de tratamiento con tenofovir y cuatro con tenofovir para la PTMF. De los 217 bebés nacidos vivos expuestos, 181 (83,4 %) recibieron la vacuna contra el VHB al nacer, 160 (88,4 %) de ellos en las primeras 24 horas de vida. En la visita de seguimiento a los nueve meses, solo uno de los 134 lactantes analizados dio positivo en la prueba del VHB.

**Conclusión:**

Esta intervención dirigida por enfermeras demuestra la viabilidad de integrar la PTMF del VHB en los departamentos de asistencia prenatal existentes, lo que es esencial para la aplicación de la iniciativa de triple eliminación. La vacunación universal al nacer es fundamental para lograr la erradicación del VHB.

## ملخص

الغرض تجربة تدخل للوقاية من انتقال فيروس التهاب الكبد ب (HBV) من الأم إلى الطفل (PMTCT) في وحدة رعاية ما قبل الولادة والأمومة في مابوتو، موزامبيق، خلال الفترة من 2017 إلى 2019.

الطريقة قمنا بتضمين فيروس التهاب الكبد ب في برنامج الفحص الحالي (لفيروس نقص المناعة البشرية (HIV) والزهري) للنساء الحوامل في زيارة الاستشارة الأولى، وتابعنا ثنائيات الأم والطفل حتى 9 أشهر بعد الولادة. قمنا بإحالة النساء اللاتي ثبتت إصابتهن بمستضد التهاب الكبد ب السطحي (HBsAg) بهدف إجراء المزيد من الاختبارات، بما في ذلك مستضد التهاب الكبد ب هـ (HBeAg) والحمل الفيروسي بفيروس التهاب الكبد ب. وفقًا للنتائج، اقترحنا عقار Tenofovir (تينوفوفير) لصحتهم أو لمنع انتقال الفيروس من الأم إلى الطفل. قمنا بإعطاء جرعة الولادة من لقاح فيروس التهاب الكبد ب، وقمنا بتقييم حالة الإصابة بفيروس التهاب الكبد ب في سن 9 أشهر.

النتائج من بين 6775 امرأة تم فحصها، كانت 270 (4.0%) نتيجتهن إيجابية للإصابة بمستضد التهاب الكبد السطحي (HBsAg)؛ في أولئك اللاتي كانت البيانات متاحة لهن، وكانت 24/265 (9.1%) إيجابية للإصابة بمستضد التهاب الكبد ب هـ، و14/267 (5.2%) كان لديهن حمل فيروسي > 200000 وحدة دولية/مل. ثمانية وتسعون (36.3%) من النساء ذوات النتيجة الإيجابية للإصابة بمستضد التهاب الكبد السطحي، كن مصابات جميعهن بفيروس نقص المناعة البشرية، بينما 97 منهن كن يتلقين العلاج المضاد للفيروسات القهقرية مع تينوفوفير. من بين النساء ذوات النتائج السلبية للإصابة بفيروس نقص المناعة البشرية، كان لدى أربع منهن دواع للعلاج بعقار تينوفوفير، وأربع لعقار تينوفوفير للوقاية من انتقال الفيروس من الأم إلى الطفل. من بين 217 طفلًا مولودًا على قيد الحياة، تلقى 181 (83.4%) جرعة الولادة من لقاح التهاب الكبد ب، و160 (88.4%) منها كانت قبل 24 ساعة بعد الولادة. في المتابعة التي استمرت على مدى 9 أشهر، كان واحد فقط من أصل 134 رضيعًا، مصابًا بفيروس التهاب الكبد ب.

الاستنتاج إن التدخل الخاص بنا، والذي يتم بقيادة الممرضة، يسلط الضوء على جدوى دمج منع انتقال فيروس التهاب الكبد ب من الأم إلى الطفل، في أقسام الرعاية السابقة للولادة، وهو أمر ضروري لتنفيذ مبادرة الإقصاء الثلاثي. يعتبر التطعيم الشامل عند الولادة أمرًا أساسيًا لتحقيق القضاء على فيروس التهاب الكبد ب.

## 摘要

目的 旨在于 2017–2019 年在莫桑比克马普托试点运行一项在产前保健和产科服务时预防乙型肝炎病毒 (HBV) 母婴传播 (PMTCT) 的干预措施。

方法 我们在孕妇第一次询诊时将 HBV 纳入当前的筛查计划【包括人体免疫缺损病毒 (HIV) 和梅毒】中,并在婴儿出生 9 个月后追踪产妇和婴儿的情况。我们将乙肝表面抗原 (HBsAg) 检测阳性的孕妇转诊接受进一步的检查,包括乙型肝炎病毒 e 抗原 (HBeAg) 和 HBV 病毒载量。根据检查结果,我们会建议服用替诺福韦,这既是出于孕妇自身的健康考虑,也是出于预防母婴传播 (PMTCT) 考虑。我们给新生儿注射出生剂量的 HBV 疫苗,并评估他们在 9 个月时的 HBV 状态。

结果在筛查的 6775 名女性中,270 (4.0%) 呈 HBsAg 阳性;在拥有可用数据的女性中,24/265 (9.1%) 呈 HBeAg 阳性,且 14/267 (5.2%) 的病毒载量 > 200 000 IU/mL。98 (36.3%) 位 HBsAg 阳性女性同时感染 HIV,其中 97 位女性正在服用替诺福韦进行抗逆转录病毒治疗。在 HIV 阴性的女性中,4 人具有替诺福韦治疗指征,4 人具有替诺福韦母婴传播预防指征。在 217 位出生时暴露的婴儿中,181 (83.4%) 人接种了出生剂量的 HBV 疫苗,其中 160 (88.4%) 人是在出生后 24 小时内接种的。在 9 个月随访时,134 位婴儿中只有一人 HBV 呈阳性。

结论 我们由护士主导的干预措施重点强调了将 HBV 的母婴传播预防纳入当前的产前保健部门的可行性,这是实施三重消除举措的基础。出生剂量的疫苗接种是实现消除 HBV 的关键所在。

## Резюме

**Цель:**

На экспериментальной основе внедрить меры по профилактике передачи вируса гепатита В (HBV) от матери ребенку (ППМР) в дородовом и родильном отделении в г. Мапуту, Мозамбик, в течение 2017–2019 гг.

**Методы:**

Авторы включили HBV в существующую программу скринингового обследования (на вирус иммунодефицита человека (ВИЧ) и сифилис) беременных женщин во время их первой консультации и наблюдали за парами «мать-ребенок» на протяжении 9 месяцев после родов. Женщин с положительным результатом на поверхностный антиген вируса гепатита B (HBsAg) направляли на дальнейшее обследование, в том числе на определение HBe-антигена вируса гепатита В (HBeAg) и вирусную нагрузку HBV. По результатам обследований авторы предложили применение препарата «Тенофовир» для лечения или ППМР. Дозу вакцины против HBV вводили младенцам при рождении, а затем оценивали HBV-статус ребенка на протяжении 9 месяцев.

**Результаты:**

Из 6775 женщин, прошедших скрининговое обследование, 270 (4,0%) имели положительный результат теста на HBsAg; среди тех, по которым имелись доступные данные, 24/265 (9,1%) имели положительный результат теста на HBeAg и 14/267 (5,2%) имели вирусную нагрузку > 200 000 МЕ/мл. Девяносто восемь женщин (36,3%) с положительным результатом теста на HBsAg были ВИЧ-инфицированными, 97 из которых получали антиретровирусную терапию тенофовиром. Среди женщин с отрицательным диагнозом ВИЧ четыре имели показания для лечения тенофовиром, а четыре — для ППМР. Из 217 контактных живорожденных младенцев 181 (83,4%) получил вакцину против HBV при рождении, 160 (88,4%) из них менее чем через 24 часа после рождения. В период 9-месячного последующего наблюдения только у одного из 134 протестированных младенцев обнаружен вирус гепатита В.

**Вывод:**

Меры с участием младшего медицинского персонала подчеркивают возможность практического внедрения ППМР HBV в работу существующих дородовых отделений и имеют важнейшее значение для реализации инициативы «тройной элиминации». Универсальная вакцинация дозой, вводимой ребенку при рождении, — это ключ к элиминации вируса гепатита B.

## Introduction

Hepatitis B virus (HBV) remains a public health threat worldwide, affecting 240 million people and causing an estimated 650 000 deaths per year.^1^ The risk of developing chronic HBV infection is strongly linked to the age at exposure, varying from approximately 90% in infants (aged < 1 year), to 50% in toddlers (aged 1–2 years) and young children (aged 3–5 years), to 5% in adults (aged ³ 18 years).^2^^,^^3^ HBV prevalence is estimated at 6.1% (95% confidence interval, CI: 4.6–8.5) among adults in sub-Saharan countries.^4^ Major gaps exist, including low birth-dose coverage (10%), low diagnosis of cases (10%) and poor access to treatment.^5^^,^^6^ The reported occurrence of mother-to-child transmission (MTCT) of HBV is lower in Africa than in Asia, partly as a result of the difference in hepatitis B e antigen (HBeAg) prevalence and genotype distribution.^7^^,^^8^ Nevertheless, a literature review estimated that 1% of newborns are infected with HBV annually, representing 367 250 newborns in sub-Saharan countries.^9^

Antenatal care provides a key opportunity for prevention of MTCT (PMTCT). HBV transmission risk is related to the maternal presence of HBeAg and to high HBV viral load (> 200 000 international units, IU per mL).^10^^,^^11^ On the identification of women with a high risk of transmission, antiviral therapy can be initiated. Already part of human immunodeficiency virus (HIV) treatment in most countries, tenofovir is the preferred recommended regimen for HBV treatment and PMTCT.^12^^,^^13^

In 2016, the World Health Organization (WHO) endorsed a global strategy to eliminate viral hepatitis as a public threat by 2030, including reducing the prevalence of hepatitis B surface antigen (HBsAg) among children to 0.1%.^14^ In addition to the scale-up of HBV vaccination as part of the Expanded Program on Immunization (EPI), birth-dose vaccination has been recommended since 2004 to protect infants from vertical and horizontal transmission. Nevertheless, global coverage remains low, especially in low-resource settings.^4^ The triple elimination initiative of WHO recommends addressing the elimination of HIV, syphilis and HBV MTCT simultaneously. An integrated approach to these three preventable diseases would be an efficient use of resources and would improve the impact in existing antenatal care and HIV PMTCT structures.^15^^–^^17^

Despite implementation of the triple elimination initiative in some countries, progress remains slow in sub-Saharan Africa.^5^ In Mozambique, HBsAg prevalence data are scarce and vary between 4.5% in women donating blood and 12.2% in young HIV coinfected adults.^18^^–^^20^ HBV vaccination has been part of the EPI since 2004, but birth-dose vaccination, recently included in the National Hepatitis Guideline, has not yet been implemented.^21^ In 2019, HIV prevalence among adult women nationwide was estimated at 15.2% and syphilis prevalence at 2.0%.^22^^,^^23^ In 2017, HIV and syphilis PMTCT interventions were implemented by the health ministry in Mozambique, but not HBV PMTCT. 

Since November 2017, the health ministry and *Médecins Sans Frontières* have been piloting an HBV PMTCT intervention with the aim of reducing the prevalence of HBV in newborns. We included HBV in the existing screening programme (for HIV and syphilis) for pregnant women at their first consultation between November 2017 and September 2019, and followed mother and child until 9 months after delivery. We describe the prevalence of HBsAg among women attending the antenatal care and maternity unit, and the cascade of care for HBsAg-positive mothers. We anticipate that this description of our pilot intervention will inform national programmes and policies, and will support the adoption of the triple elimination strategy by Mozambique and other low-income countries.

## Methods

### Setting

Our HBV PMTCT project was conducted in the antenatal care and maternity unit of Chamanculo General Hospital in Maputo, the capital city of Mozambique. 

### Study design

We retrospectively analysed the project data from pregnant women diagnosed as HBsAg positive and their newborns. We hired two nurses to perform initial and follow-up consultations, as well as the tracing of HBsAg-positive women and their infants. We provided training to all health ministry and project nurses and midwives on HBV screening, PMTCT and vaccination, either in the form of a one-day theory course or on-the-job training. We developed original educational material for patients. A clinician provided medical supervision, and we adapted existing registers to include the HBV component. All care was provided free of charge.

### Screening

Nurses collected capillary blood from women who gave their consent, and rapid diagnostic tests were performed: SD Bioline Syphilis 3.0, Determine HBsAg 2 and Alere Determine HIV-1/2 (Abbott Diagnostics Korea, Inc., Giheung-gu, Republic of Korea) followed, if positive, by Uni-Gold HIV (Trinity Biotech Public Limited Co., Bray, Ireland).

We proposed HBV vaccination (Hepatitis B Vaccine rDNA, Serum Institute of India Pvt. Ltd, Pune, India) to women who tested HBsAg negative; antenatal care nurses administered three doses of vaccination onsite at admission, month 1 and month 2.

### HBsAg-positive care

We referred women with a positive HBsAg test to the project nurses for counselling and further tests. The project nurses performed hepatitis C serology (Bioline HCV, Abbott Diagnostics Korea) onsite, and sent full blood samples to a local private laboratory to determine HBV viral load (cobas® 6800 system, Roche Diagnostics, Burgess Hill, England) and conduct tests for HBeAg (MiniVidas®, BioMerieux, Marcy-l’Étoile, France), liver function, creatinine and full blood count. From July 2019, laboratory technicians determined HBV viral load at a *Médecins Sans Frontières* clinic using Xpert® HBV Viral Load (Cepheid AB, Solna, Sweden), providing same-day results. 

Following WHO recommendations, nurses initiated tenofovir treatment at 300 mg per day if there was a suspicion of cirrhosis based on the aspartate-aminotransferase-to-platelet ratio index (APRI) with a score of > 2 in adults, or persistently abnormal alanine aminotransferase and HBV viral load of > 20 000 IU/mL in those aged > 30 years.^3^ Following European Association for the Study of the Liver recommendations, nurses initiated tenofovir PMTCT if HBV viral load was > 200 000 IU/mL from 28 weeks of pregnancy.^12^ We proposed tenofovir PMTCT for up to 4 weeks after delivery, with its continuation assessed on an individual basis. For HIV coinfected women, tenofovir was included as part of antiretroviral treatment (ART) following national guidelines.^24^

### Follow-up

We integrated HBV follow-up into the antenatal care routine consultation flow. Women who missed appointments were traced by phone, and women attending the maternity unit at the time of delivery were screened by midwives. Midwives gave the birth-dose HBV vaccine to exposed newborns as soon as possible after birth, and referred newborns to EPI for routine vaccination. We followed mothers and infants for 9 months after birth, and determined the infant’s HBV status by HBsAg status and HBV viral load.

At the end of the 9-month follow-up period, we referred HBV mono-infected women and positive infants to the Central Hospital of Maputo, and HBV–HIV coinfected women to the national HIV programme.

### Data processing

During consultations and follow-up, project nurses used questionnaires to collect routine individual demographic data from HBsAg-positive women enrolled in HBV care, and entered standardized clinical files in a secure and anonymized database.^25^

The main outcome of our project was the HBV status of the infant at 9 months. We recorded data on HBV PMTCT eligibility and HBV vaccination at birth (verified by vaccination card). We compared baseline characteristics by maternal follow-up status (those who adhered to the care protocol, including those who completed the programme or stopped for other reasons, with those lost to follow-up) using χ^2^ or Fisher’s exact test for categorical variables, and Student *t*-test for continuous variables. We performed data analysis using STATA version 13.1 (STATACorp, College Station, United States of America).

### Data sharing

According to the data-sharing policy of *Médecins Sans Frontières*, the underlying data set is available upon request.

### Ethics statement

This pilot project received official approval from the Health Director of Maputo City. Our analysis was based on routinely collected data without identifiable information, and fulfilled the exemption criteria set by the *Médecins Sans Frontières* Ethics Review Board.

## Results

**Fig. 1:**
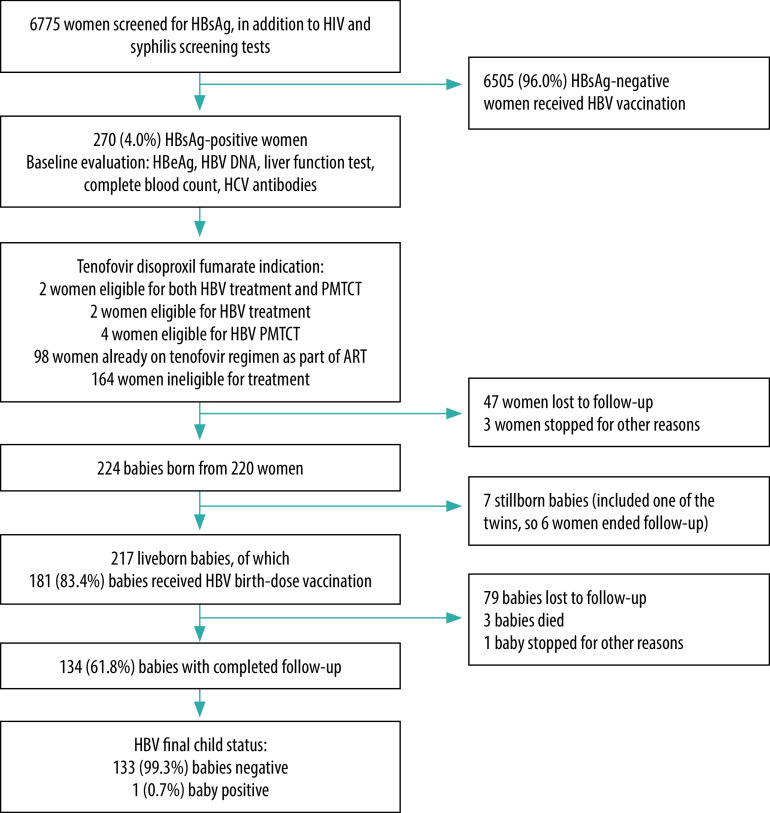
Flow and intervention for HBV-screened pregnant women, antenatal care and maternity unit of Chamanculo Hospital, Mozambique, 2017–2019

ART: antiretroviral therapy; HBeAg: hepatitis B e antigen; HBsAg: hepatitis B surface antigen; HBV: hepatitis B virus; HBV DNA: viral load of hepatitis B virus; HCV: hepatitis C virus; HIV: human immunodeficiency virus; PMTCT: prevention of mother-to-child transmission.

Between 11 November 2017 and 30 September 2019, we screened 6775 women for HBsAg and 270 (4.0%) tested positive. A total of 47 (17.4%) were lost to follow-up before delivery and a further three women (1.1%) stopped their follow-up for various reasons (e.g. miscarriage, moving to a new location and therefore a new health facility) after discussion with the medical team. The remaining 220 women gave birth to 217 live babies and seven stillborns (Fig. 1). We registered four multiple pregnancies, including one of the stillborns. A further 79 women (29.3%) were lost to follow-up, another left the programme during postpartum follow-up after discussion with the medical team, and three babies died during follow-up (date of death unknown). A total of 134 babies (61.8%) completed the 9-month follow-up.

### Baseline assessment 

The baseline characteristics of the 270 HBsAg-positive women were similar between those who adhered to the care protocol and those who were lost to follow-up, with one exception: women who were lost to follow-up were more likely to be HIV-positive than women who completed follow-up (*P* = 0.005; Table 1). Among HBsAg-positive women, the median age was 29.1 years (interquartile range, IQR: 23.5–33.1) at admission (Table 1). Most women (70.7%; 191/270) attended their first antenatal care consultation during the second trimester of pregnancy, followed by 13.3% (36/270) during the third trimester and 10.4% (28/270) during the first trimester. At the maternity unit, 13 patients (4.8%) screened positive £24 hours after delivery and one screened positive > 24 hours after delivery (0.4%).

**Table 1:** Sociodemographic and medical characteristics of HBsAg-positive women, antenatal care and maternity unit of Chamanculo Hospital, Mozambique, 2017–2019

Characteristic	No. (%)			*P*
	Screened positive for HBsAg (*n* = 270)^a^	Adhered to care protocol (*n* = 144)^a^	Lost to follow-up (*n* = 126)^a^	
**Mozambican nationality**	269 (99.6)	144 (100.0)	125 (99.2)	0.286
**Employment**				0.157
Housework	162 (60.0)	77 (53.5)	85 (67.5)	
Employed	75 (27.8)	46 (31.9)	29 (23.0)	
Student	24 (8.9)	16 (11.1)	8 (6.3)	
Other	9 (3.3)	5 (3.5)	4 (3.2)	
**Marital status**				0.115
Living together	187 (69.3)	93 (64.6)	94 (74.6)	
Single	65 (24.1)	41 (28.5)	24 (19.0)	
Married	13 (4.8)	9 (6.3)	4 (3.2)	
Other	5 (1.9)	1 (0.7)	4 (3.2)	
**Previous HBV vaccination**	2 (0.7)	1 (0.7)	1 (0.8)	0.364
**Previous HBV treatment**	0 (0.0)	0 (0.0)	0 (0.0)	NA
**HIV positive^b^**	98 (36.3)	41 (28.5)	57 (45.2)	0.005
**Other comorbidities**				
Tuberculosis	0 (0.0)	0 (0.0)	0 (0.0)	NA
Hypertension	2 (0.7)	1 (0.7)	1 (0.8)	0.929
Non-insulin dependent diabetes	2 (0.7)	1 (0.7)	1 (0.8)	0.929
Renal insufficiency	0 (0.0)	0 (0.0)	0 (0.0)	NA
**Status at first consultation**				0.197
Pregnant	255 (94.4)	136 (94.4)	119 (94.4)	
1st trimester	28 (10.4)	18 (12.5)	10 (7.9)	
2nd trimester	191 (70.7)	102 (70.8)	89 (70.6)	
3rd trimester	36 (13.3)	16 (11.1)	20 (15.9)	
≤ 24 hours post-delivery	13 (4.8)	8 (5.6)	5 (4.0)	
> 24 hours post-delivery	1 (0.4)	0 (0.0)	1 (0.8)	
Not known	1 (0.4)	0 (0.0)	1 (0.8)	

HBsAg: hepatitis B surface antigen; HBV: hepatitis B virus; HIV: human immunodeficiency virus; NA: not applicable.

^a^ Median age (interquartile range) of women who screened positive for HBsAg, adhered to care protocol and were lost to follow-up was 29.1 (23.5–33.1), 28.1 (22.8–32.6) and 29.95 (24.7–33.3) years, respectively. Age was not significant (*P* = 0.079).

^b^ See Table 3 for details of infection stage and antiretroviral regime.

A total of 9.1% (24/265) of the women tested HBeAg positive (Table 2). Viral load was undetectable for 36.7% (98/267), < 20 000 IU/mL for 54.7% (146/267), 20 000–200 000 IU/mL for 3.4% (9/267) and > 200 000 IU/mL for 5.2% (14/267).

**Table 2:** Biological characteristics at first consultation of HBsAg positive women, antenatal care and maternity unit of Chamanculo Hospital, Mozambique, 2017–2019

Biological characteristic	No. with biological characteristic/no. for whom data were available^a,b^ (%)			*P*
	Screened positive for HBsAg	Adhered to care protocol	Lost to follow-up	
**HBeAg positive**	24/265 (9.1)	13/142 (9.2)	11/123 (8.9)	0.779
**HBV viral load, IU/mL**				0.583
Undetectable	98/267 (36.7)	47/144 (32.6)	51/123 (41.5)	
≤20 000	146/267 (54.7)	85/144 (59.0)	61/123 (49.6)	
> 20 000–200 000	9/267 (3.4)	5/144 (3.5)	4/123 (3.3)	
> 200 000	14/267 (5.2)	7/144 (4.9)	7/123 (5.7)	
**APRI category^c^**				0.540
< 1	258/265 (97.4)	141/144 (97.9)	117/121 (96.7)	
1–2	6/265 (2.3)	3/144 (2.1)	3/121 (2.5)	
> 2	1/265 (0.4)	0/144 (0.0)	1/121 (0.8)	
**ALT > 40 IU/mL**	9/268 (3.4)	8/143 (5.6)	1/125 (0.8)	0.890

ALT: alanine aminotransferase; APRI: aspartate-aminotransferase-to-platelet ratio index; HBeAg: hepatitis B e antigen; HBsAg: hepatitis B surface antigen; HBV: hepatitis B virus; IU: international unit.

^a^ Note that data were not available for all 270 women who screened positive for HBsAg.

^b^ Note that data may be subject to rounding errors.

^c^ Median value of APRI (interquartile range) for women who screened positive for HBsAg, adhered to care protocol and were lost to follow-up was 0.29 (0.21–0.38), 0.28 (0.22–0.37) and 0.30 (0.19–0.42) years, respectively. APRI score, when tested as a continuous variable using the Student *t*-test, was not significant (*P* = 0.088).

**Fig. 2:**
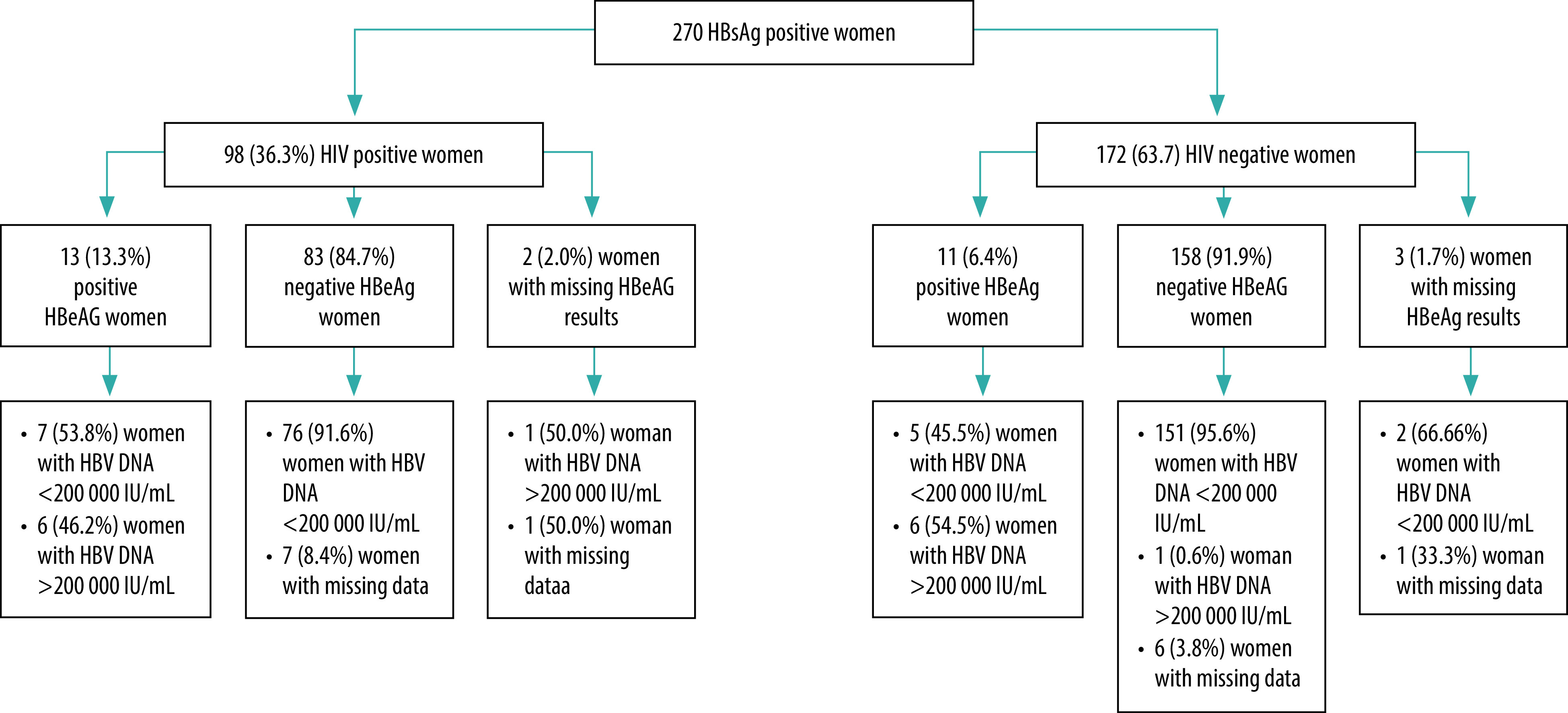
Biological status of HBV-screened pregnant women at enrolment, antenatal care and maternity unit of Chamanculo Hospital, Mozambique, 2017–2019

HBeAg: hepatitis B e antigen; HBsAg: hepatitis B surface antigen; HBV: hepatitis B virus; HBV DNA: viral load of hepatitis B virus; HIV: human immunodeficiency virus; IU: international unit.

Screening revealed that 36.3% (98/270) of the women were coinfected with HIV (Table 3). The percentage testing positive for HBeAg was non-significantly higher among HIV-positive women (13/98; 13.3%) than HIV-negative women (11/(270–98); 6.4%; *P* > 0.05). Among the HBeAg-positive women, 46.2% (6/13) of HIV-positive women had a viral load above the PMTCT threshold compared with 54.5% (6/11) of HIV-negative women (*P* > 0.05; Fig. 2). Among the HBeAg-negative women, no HIV-positive women with viral load known had a viral load above the PMTCT threshold, and only one (out of 158; 0.6%) HIV-negative woman had a viral load above the PMTCT threshold.

**Table 3:** HIV infection stage and ART regime of HBsAg–HIV-positive women, antenatal care and maternity unit of Chamanculo Hospital, Mozambique, 2017–2019

Variable	No. (%)			*P*
	Screened positive for HBsAg and HIV (*n* = 98)	Adhered to care protocol (*n* = 41)	Lost to follow-up (*n* = 57)	
**Stage of HIV infection**				0.123
WHO 1	87 (88.8)	40 (97.6)	47 (82.5)	
WHO 2	3 (3.1)	0 (0.0)	3 (5.3)	
WHO 3	2 (2.0)	0 (0.0)	2 (3.5)	
Not known	6 (6.1)	1 (2.4)	5 (8.8)	
**ART regime**				1.000
Tenofovir / lamivudine / efavirenz	97 (99.0)	41 (100.0)	56 (98.2)	
Other	1 (1.0)	0 (0.0)	1 (1.8)	
**Time on ART, months**				0.276
< 6	56 (57.1)	21 (51.2)	35 (61.4)	
> 6	39 (39.8)^a^	19 (46.3)^a^	20 (35.1)^a^	
Not known	3 (3.1)	1 (2.4)	2 (3.5)	

ART: antiretroviral treatment; HBsAg: hepatitis B surface antigen; HIV: human immunodeficiency virus; WHO: World Health Organization.

^a^ Median time on ART (interquartile range) for HIV-positive women who screened positive for HBsAg, adhered to care protocol and were lost to follow-up was 3.77 (1.51–6.54), 3.85 (2.79–5.70) and 3.77 (2.02–5.71) years, respectively.

Fibrosis was assessed using the APRI score: out of the 265 women assessed, one (0.4%) woman had an APRI score of > 2, and six (2.3%) an APRI score of 1–2. A total of nine women (3.4%; 9/268) had an abnormal alanine aminotransferase level (> 40 IU/mL; Table 2).

### HBV treatment

All 98 HIV coinfected HBsAg-positive women received tenofovir-based ART, except one woman screened in peripartum who was on an azidothymidine regimen; 39 had been on tenofovir disoproxil fumarate treatment for > 6 months (Table 3).

Among the 172 HIV-negative pregnant women, only four (2.3%) had an indication for treatment for their own health according to the WHO recommendation; of these, two also had a viral load of > 200 000 IU/mL. Three women initiated treatment and one was lost to follow-up. Another four women (2.3%) had an indication for PMTCT with a viral load of > 200 000 IU/mL; two initiated tenofovir treatment, one was lost to follow-up and one delivered before initiation. During the 9-month follow-up, no additional women were eligible for tenofovir treatment.

### HBV-exposed infants

For the 217 liveborn babies, the median birth weight was 3.1 kg (IQR: 2.9–3.5) and 111 (51.2%) were female. Of these, 181 (83.4%) received birth-dose vaccination at the first contact: 160 (88.4%) babies were vaccinated < 24 hours and 13 (7.2%) ³ 24 hours after birth. The birth-dose vaccination was administered in < 24 hours for 80% (4/5) of babies born at home and for 75.4% (150/199) of babies born in a health facility (Table 4).

**Table 4:** Timeliness of HBV birth-dose vaccine by place of birth, for HBV-exposed babies born alive to HBsAg-positive mothers, antenatal care and maternity unit of Chamanculo Hospital, Mozambique, 2017–2019

Birth-dose vaccination	No. (%)				*P*
	Health facility (*n* = 199)	Home (*n* = 5)	Unknown (*n* = 13)	Total (*n* = 217)	
**Time of birth-dose vaccine after birth, hours**					0.248
< 4	100 (50.3)	3 (60.0)	6 (46.2)	109 (50.2)	
4–23	50 (25.1)	1 (20.0)	0 (0.0)	51 (23.5)	
24–47	4 (2.0)	0 (0.0)	1 (7.7)	5 (2.3)	
≥ 48	7 (3.5)	1 (20.0)	0 (0.0)	8 (3.7)	
Not known	8 (4.0)	0 (0.0)	0 (0.0)	8 (3.7)	
**Not vaccinated**	30 (15.1)	0 (0.0)	6 (46.2)	36 (16.6)	1.000

### Outcomes

Of the 270 women who tested positive for HBsAg, 144 (53.3%) women initially adhered to the care protocol and 126 (46.7%) were lost to follow-up. Of the women adhering to the protocol, 131 (48.5%) completed the full follow-up, nine (3.3%) lost their child and four (1.5%) stopped for other reasons after discussion with the medical team. Routine EPI HBV vaccination was completed for all infants with 9-month outcome. Of 134 infants tested at 9 months, one was positive for HBsAg. The mother screened positive for HIV and HBV at 28 weeks of pregnancy, with a positive HBeAg result and a high HBV viral load (220 000 000 IU/mL). After immediate initiation of tenofovir-based ART (on the same day as the screening result), the viral load decreased but was still high around the time of delivery (79 600 000 IU/mL). The mother missed several follow-up appointments. Born in another health centre, the baby was not vaccinated at birth; despite three later EPI vaccine doses, the baby was HBsAg positive with detectable viral load (20 800 IU/mL) at the age of 1 year. The HIV status of the infant was unknown at the last consultation.

### Cost

To implement the project, the additional commodities cost 44.53 United States dollars per HBsAg-positive woman (Box 1).

Box 1 **Commodity cost of HBV activities in the antenatal care and maternity unit of Chamanculo Hospital, Mozambique, 2017–2019**

- HBsAg test: US$ 1.62
- HBeAg test (private laboratory): US$ 15.37
- Xpert® HBV Viral Load cartridge:^a^ US$ 26.01
- Complete blood count: US$ 0.30
- Aspartate aminotransferase and alanine aminotransferase: US$ 0.14
- Creatinine test: US$ 0.20
- HBV vaccine (for adult): US$ 0.56
- HBV birth-dose vaccine: US$ 0.33
- Total cost of commodities: US$ 44.53

HBeAg: hepatitis B e antigen; HBsAg: hepatitis B surface antigen; HBV: hepatitis B virus; US$: United States dollars.^a^ Alternative determination of HBV viral load by private laboratory would incur a cost of US$ 81.31.

## Discussion

Our observed HBsAg prevalence was below the published prevalence in Mozambique. Based on the few previous studies and country indicators, a study reported a modelled estimate of HBsAg prevalence at 7.5% (95% CI: 5.6–8.7) in 2016.^20^ The difference may be related to the different performance of the screening tests^26^ or to the specific screened populations. Further, most of the publications including information on HBsAg prevalence were focused on HIV-positive patients or at-risk groups.^19^^,^^27^^,^^28^ We did not find any previous serology survey in antenatal care in Mozambique. Despite the low risk of adults becoming chronic carriers and the negligible impact of catch-up vaccination of adults on the population prevalence,^29^ we proposed HBV vaccination to HBsAg-negative mothers; our reason for doing so was to avoid infection for potential future pregnancies, considering the high national fertility rate (4.85 births per woman in 2018).^30^

We identified only 5.2% of women with viral load > 200 000 IU/mL and 9.1% with positive HBeAg. The low HBeAg prevalence was expected, as already reported in the literature.^20^^,^^31^ Initiation of tenofovir PMTCT based on HBeAg positivity, an indicator of high risk of transmission when viral load data are unavailable, was recently recommended by WHO.^32^ Despite concerns about performance, an HBeAg rapid diagnostic test already exists.^33^ However, if we had used an HBeAg-based algorithm in our cohort, we would have treated 5/11 HBeAg-positive/HIV-negative women with viral load < 200 000 IU/mL unnecessarily, and would have missed one HBeAg-negative woman with viral load of > 200 000 IU/mL. This finding corresponds to a sensitivity and a specificity of HBeAg to identify HBV viral load of > 200 000 IU/mL of 85.7% (6/7) and 96.8% (151/156), respectively, for HIV-negative women, and 100% (6/6) and 91.6% (76/83) for HIV-positive women. A recent meta-analysis of pregnant women without concurrent ART reported similar results: a pooled sensitivity of 88.2% (95% CI: 83.9–91.5) and specificity of 92.6% (95% CI: 90.0–94.5).^11^

Among the HIV-negative women, tenofovir was indicated in only four women for treatment for their own health and four women for PMTCT. Fluctuations of HBV viral load or aspartate or alanine aminotransferase during pregnancy and postpartum may influence the decision for treatment.^34^ We referred women to a hepatology department to ensure long-term follow-up. The low percentage of women needing treatment is similar to that observed in the Prevention of Liver Fibrosis and Cancer in Africa (PROLIFICA) study in West Africa, but lower than the 11% reported by a feasibility study in the Democratic Republic of the Congo.^6^^,^^35^^,^^36^ Nevertheless, related to the national HIV prevalence, our intervention identified a high proportion of HIV coinfected women, most of them already on a tenofovir-based regimen.

Universal birth-dose vaccination, cheap and accessible, is already recognized as crucial to fight HBV transmission in early childhood.^37^ In Mozambique, birth-dose vaccination is not routinely implemented. In our project, none of the children who received the birth-dose vaccination was infected. The only HBsAg-positive child did not receive the birth-dose; although the HIV coinfected mother initiated ART during pregnancy, she continued with high HBV viral load during follow-up.

Our study benefited from a comprehensive approach to HBV care in a routine antenatal facility, with the collaboration of health ministry personnel. This collaboration led to integrated HBV care and prevention in the new National Hepatitis and Triple Elimination Guidelines.^38^^,^^39^ The Global Fund included procurement for HBV screening tests in 2020, and for tenofovir in the 2021–2023 grant, to implement the triple elimination strategy. *Médecins Sans Frontières* currently supports the health ministry to finalize a training package and an implementation plan to scale up the triple elimination strategy in the country. HBV birth-dose vaccines will soon be available, despite delay due to the coronavirus disease 2019 pandemic.

Our study had several limitations. We piloted our intervention under real conditions; however, this meant that routine programmatic data were prone to suboptimal quality and completeness, and other information (collected by interview) was subject to recall bias. No data on HBsAg-negative mothers were collected, preventing the possibility of identifying risk factors for HBsAg positivity. The health ministry reported HIV and syphilis prevalence of 5% and 2% for the same period. However, data on retesting and known HIV-positive women were not separated, leading to different screened populations. In addition, no data were collected on HIV and syphilis routine care.

Just over half of the women included in our pilot completed follow-up, despite individual tracing. MTCT may be underestimated and vaccination coverage overestimated, as women lost to follow-up may be less compliant. HIV prevalence was higher among the women who were lost to follow-up. Retention in care has been identified as a challenge for HIV PMTCT in Mozambique. Despite high screening coverage and access to treatment (99% for both), a report found 65% retention at 12 months in women starting ART during pregnancy in 2018, leading to high HIV transmission to children.^40^ Qualitative studies have identified the main factors affecting retention in HIV care: social stigma and health system inadequacies.^41^^–^^43^ In our project the care flow and sites were different for HBV and HIV, and women may have prioritized HIV follow-up, with tenofovir already included in ART. Furthermore, treatment was not initiated for three HIV-negative women who were eligible for HBV treatment or PMTCT but lost to follow-up or screened late. Integrating HBV care into existing circuits in antenatal care, together with an HIV–syphilis package, family planning and intimate partner violence counselling, will facilitate adherence.

Finally, we did not perform a cost analysis of the project. Nevertheless, the additional cost of integrating the HBV intervention into the existing flow would be limited. HIV–HBV coinfected women are already enrolled in an HIV programme. For HIV-negative women, health ministry nurses and midwives could include PMTCT of HBV and vaccination in their routine practice. The commodity cost would also decrease with the HBeAg algorithm, with new lower-cost rapid diagnostic tests and birth-dose vaccines to be supplied from 2022 by Gavi, the Vaccine Alliance.

Universal birth-dose vaccination should be a priority for the country. Access to compact prefilled HBV vaccines outside of cold chain and the involvement of community actors are key considering the large number of home deliveries, especially in non-urban settings.^44^^–^^46^ Decentralization and scale-up of HBV activities would be feasible at the different health structure levels, if access to tests, vaccines and treatment could be ensured.^47^ The development of point-of-care tests is still needed (e.g. HBeAg, HBV viral load, HBV core antigen).^48^^,^^49^ An HBeAg-based algorithm, already included in the national HBV protocol, would facilitate PMTCT of HBV projects in the absence of HBV viral load data.

Our results demonstrate the feasibility of a nurse-led intervention providing HBV PMTCT, including birth-dose vaccination; we have also demonstrated that such activities can be integrated with existing screening programmes to provide a single PMTCT of HBV–HIV–syphilis. With a very low proportion of women at high risk, birth-dose HBV vaccination is key to achieving the 2030 target of HBV elimination.
